# Population-based epidemiological study of cervical cancer in Khuzestan province, Iran (2014–2019)

**DOI:** 10.1186/s12889-026-26428-y

**Published:** 2026-01-31

**Authors:** Parvin Shahry, Zeinab Mohammadi khoshoei, Maria Cheraghi, Abdolhassan Talaiezadeh, Saeed Ghanbari, Shabnam Yousefi shad

**Affiliations:** 1 Department of Public Health, School of Health, Ahvaz Jundishapur University of Medical Sciences, Ahvaz, Iran; 2 Cancer, Environmental and Petroleum Pollutants Research Center, Ahvaz Jundishapur University of Medical Sciences, Ahvaz, Iran; 3 Department of General Surgery, School of Medicine, Ahvaz Jundishapur University of Medical Sciences, Ahvaz, Iran; 4 Department of Biostatistics and Epidemiology, School of Health Ahvaz, Jundishapur University of Medical Sciences, Ahvaz, Iran

**Keywords:** Cervical cancer, Five-year survival rate, Khuzestan, Iran

## Abstract

**Background:**

Cervical cancer is the third most common cancer and the fourth leading cause of deaths among women worldwide and presents a significant public health challenge, with regional variations in its impact. Understanding the epidemiology of cancer is crucial for policymakers to develop effective prevention strategies. The study provides a comprehensive analysis of the epidemiology of cervical cancer in Khuzestan province, Iran from 2014 to 2019, using data from cancer registries.

**Materials and methods:**

This retrospective study reviewed all registered cervical cancer patients in the Khuzestan province over a six-year period 2014–2019. descriptive statistics, Kaplan–Meier survival analysis, and Cox regression was used to assess the impact of demographic characteristics on patient survival, in addition, Age-standardized incidence rates were reported annually. Time series analysis was performed using Minitab to examine temporal disease trends, with the best-fitting model.

**Results:**

A total of 459 cervical cancer cases were analyzed, with a mean age of 54 (SD = 13.02) years. Among these, 91.1% were diagnosed by pathological reports, 5.2% clinically, and 2.6% through death certificates. The highest incidence rate was 2.83 per 100,000 women in 2018, and the lowest was 0.74 in 2014. The five-year survival rate for the studied cases was 65.7%.

**Conclusion:**

The incidence of cervical cancer in Khuzestan province increased during 2014–2019, although it declined compared with earlier years. Despite improvements, the five-year survival rate remains lower than other provinces. Persistent challenges include inequities in access to screening and limited availability of HPV vaccination at effective ages. Expanding screening programs, promoting early detection, and improving access to vaccination should be prioritized to reduce the disease burden.

## Background

According to the Global Cancer Observatory (GLOBOCAN) and the World Health Organization (WHO), cervical cancer is the third most common cancer and the fourth leading cause of deaths among women worldwide. In 2020, Global cancer statistics indicated that 604,127 women were diagnosed with cervical cancer, and approximately 341,831 of these women lost their lives due to the disease [1]. In 2019 cervical cancer causes 2.52 million years of life lost among young women worldwide [2]. In 2020, around 90% of new cervical cancer cases occurred in low- and middle-income countries [1], with 144,400 deaths in Asia [3]. In Asia, the highest Age-Standardized Rate(ASR) (24.5 per 100,000), was found in the Maldives, Indonesia and Myanmar. The three countries with the lowest ASR (less than 3 per 100,000) were Iraq, Iran, and Yemen [1]. Iran had an ASR of 2.3 per 100,000 women in 2020 [4]. The incidence of cervical cancer in Iran is lower than the global average, ranking 11th among female cancers [5, 6]. The mean age of patients diagnosed with cervical cancer worldwide is 52.2 years, it is 51.9 in Iran [5] is higher than several European countries (44.7 years) [7].

In Iran, screening is often conducted at the request of women or the recommendation of gynecologists or midwives, and there is no organized and active program for their follow-up. In these types of screenings, individuals are usually not comprehensively covered, leading to the diagnosis of patients at advanced stages and, consequently lower survival rates [5]. Existing research shows that organized screening programs at the population level have reduced the incidence of cervical cancer by 50 to 80% [8]. Although the incidence of cervical cancer in Iran is low, but the associated risk factors are not few, this could lead to an increase in the incidence of cervical cancer in the future [9]. Moreover, the costs imposed by cervical cancer on the healthcare system are significant in Iran, The most expensive part of gynecological cancer treatment is cervical cancer chemotherapy, with an estimated cost of $5,487,816 [10] .This amount reflects the heavy economic burden that cervical cancer imposes on the Iran healthcare system.

Khuzestan province is located in south-west Iran, with total population of 4,710,509 [11].This region is known for its diverse ethnic groups mainly Caucasians (66.4%) and Arabs (33.6%) [12]. Considering that the geographical distribution pattern of cervical cancer incidence in Iran may be related to various environmental, racial, and individual factors, and that the survival of cancer patients is influenced by factors such as lower socioeconomic status, access to healthcare services, early diagnosis etc. So far, no similar studies on cervical cancer have been observed in Khuzestan Province. Therefore, the aim of this study is to conduct an epidemiological analysis of cervical cancer in Khuzestan Province from 2014 to 2019, based on cancer registry data.

## Methods

This retrospective population-based cohort study is conducted over a six-year period (2014–2019) using data from the Khuzestan Cancer Registry-based Population at Jundishapur University of Medical Sciences, Ahvaz, Iran. The registry includes all diagnosed cancer cases. Demographic information including : (education level, employment status, ethnicity, place of residence, patient status, and time of death) was obtained over a four-month period by telephone interviews. Telephone follow-up was conducted from December 22, 2023, to March 25, 2024. Each case was contacted three to four times on different days and at varying hours until a response was obtained. For deceased patients, the date of death was confirmed using the national death registry and verified by death certificates whenever available. All calls were conducted by two trained interviewers, who received brief preparatory training on respectful communication, confidentiality, and accurate data recording to ensure consistency in data collection. Participants who could not be reached or whose survival status was unknown by the end of follow up we were treated as cencored cases. To assess the potential impact of attrition bias resulting from loss to follow-up, a sensitivity analysis was performed as recommended for studies with incomplete follow-up data. For missing survival times, an imputation approach was applied using the mean survival duration observed among deceased patients and among those alive at the end of follow-up. These imputed values were used to construct best- and worst-case scenarios to evaluate the robustness of the survival estimates.

Registered cases are classified according to the ICD-10 and ICD-O-3 standards, based on age, age group, and place of residence. According to the International Statistical Classification of Diseases,10th Revision (ICD-10), particularly code C53, which pertains to cervical cancer [13]. Cases were deduplicated, and data from non-resident patients living in neighboring provinces who sought medical care in Khuzestan province were excluded from the analysis. In order to facilitate the calculations of Age-Standardized Rates (ASR) and Crude Rates (CR), age groups have been established in specific age intervals (for example 15–19, 20–24, 25–29, 30–34, etc.).

Descriptive and survival analyses were performed using SPSS version 16 to summarize patient characteristics and estimate the five-year survival rate with the Kaplan–Meier method. To evaluate the effects of demographic characteristics on patient survival, univariate Cox regression analyses were first conducted. The effect size in this analysis was expressed as the hazard ratio (HR). Variables that were statistically significant in the univariate analyses (p-value < 0.05) were subsequently included in multivariate Cox regression models to identify independent predictors of survival.

Additionally, to examine the temporal trend of the disease, a time series analysis was performed using Minitab software. Several trend models—including linear, quadratic, exponential, and S-curve models—were tested to determine the best fit for the observed data. The Mean Absolute Percentage Error (MAPE**)** served as the main criterion for assessing model accuracy; models with lower MAPE values indicated a better fit. The model with the lowest MAPE was selected as the optimal model for describing the temporal trend of the cervical cancer.

## Results

During the 2014 and 2019 period, a total of 459 cases of cervical cancer were identified in this region. 436 cases were found to be eligible for the analysis (10 cases were recorded as deaths due to other causes, and 13 cases were from outside the province), survival and other demographic information were obtained for 303 patients through an active follow-up approach .

The mean age and standard deviation (SD) of patients at diagnosis was 54 years (SD = 13.02) with a range of 19 to 89 years. Among patients who were under active follow-up, 31.6% were illiterate, 33.7% had primary education, 17.9% had secondary education, and 16.8% had a diploma or higher. Regarding employment status, 93.5% of patients were housewives and 6.5% were employed. Arab ethnicity with 37.5%, Bakhtiari ethnicity with 25.7% and Fars ethnicity with 25.3% accounted for the most cases of patients in the province.

Regarding tumor grade, Grade 1 tumors accounted for 26% of cases, Grade 2 for 36%, and Grade 3 for 38%. Among these cases, 91.1% were diagnosed based on the pathological reports, 5.2% identified clinically, 2.6% using the death certificate, and 1.1% through cytology in the cancer registration system.

**Fig. 1:**
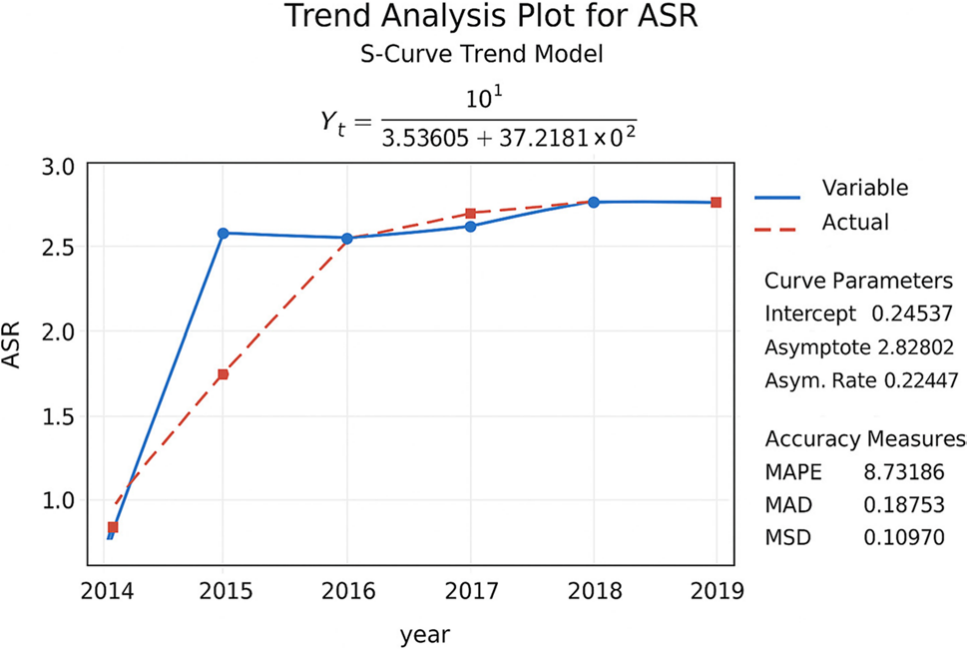
Time series trend analysis of ASR using the s-curve model of cervical cancer in Khuzestan province, Iran, from 2014 to 2019

Cancer Registry statistics indicated that the highest ASR was 2.83 per100, 000 women in 2018; and the least was 0.74 per 100,000 women in 2014 (Table 1; Fig. 1). For further investigation of the temporal trend, time series analysis was applied. Among the tested models (linear, quadratic, exponential, and S-curve), the S-Curve Trend Model demonstrated the best performance in describing the actual behavior of the ASR data. The model achieved a Mean Absolute Percentage Error (MAPE) of approximately 8.7%, indicating a high level of accuracy in fitting the observed data. Since the MAPE value is below 10%, the model provides an excellent predictive performance and a reliable representation of the temporal pattern. The analysis reveals that the overall trend of ASR is increasing, showing a clear upward trajectory over time. The model further suggests that after a period of rapid growth between 2014 and 2015, the system reached a state of stability from 2015 to 2019, implying that future variations are expected to remain minimal) (Fig. 1).

**Fig. 2:**
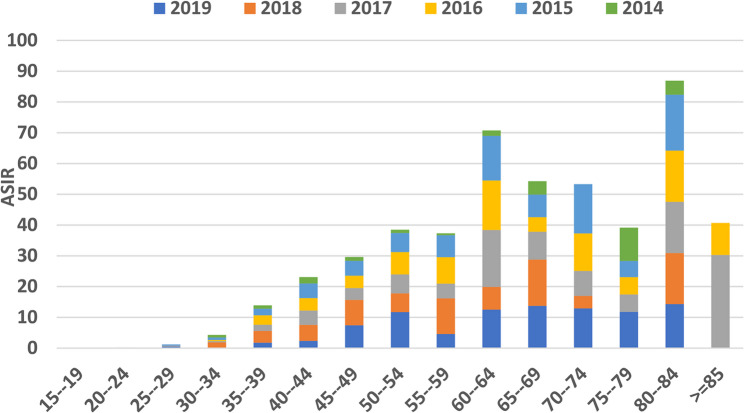
Age specific incidence rate (ASIR) (per 100,000) of cervical cancer in Khuzestan province, Iran, (2014–2019)

The age-specific incidence rates (ASIR) of cervical cancer in different age groups from 2013 to 2019 in Khuzestan province, Iran, are examined (Fig. 2). Specifically, the highest ASIR in 2015, 2016, 2018, and 2019 were reported in the age group of 80 to 84 years, with rates of 18.18, 16.63, 16.62, and 14.28 per 100,000 women (Fig. 2; Table 1), respectively. Additionally, in 2013, the highest rate (10.81 per 100,000) was found in the age group of 70 to 74 years. In 2017, the incidence rate peaked at 30.24 per 100,000 for those aged 85 years and older.

Overall, these data highlight the variations in cervical cancer incidence rates across different age groups, with a notable increase in 2017 for the older age group.

**Table 1:** Age-specific incidence rate (80 − 84years) (ASIR), crude incidence rate, and Age-standardize rate (ASR) of cervical cancer in Khuzestan Province (2014–2019)

Year	Number	Crude incidence rate (per 100 000)	Age-specific incidence rate (ASIR) (per 100 000) 80–84	ASR (per 100 000)
2014	31	0.66	4.55	0.74
2015	51	2.14	18.18	2.64
2016	51	2.20	16.63	2.62
2017	52	2.22	16.60	2.65
2018	63	2.68	16.62	2.83
2019	59	2.44	14.28	2.81

**Fig. 3:**
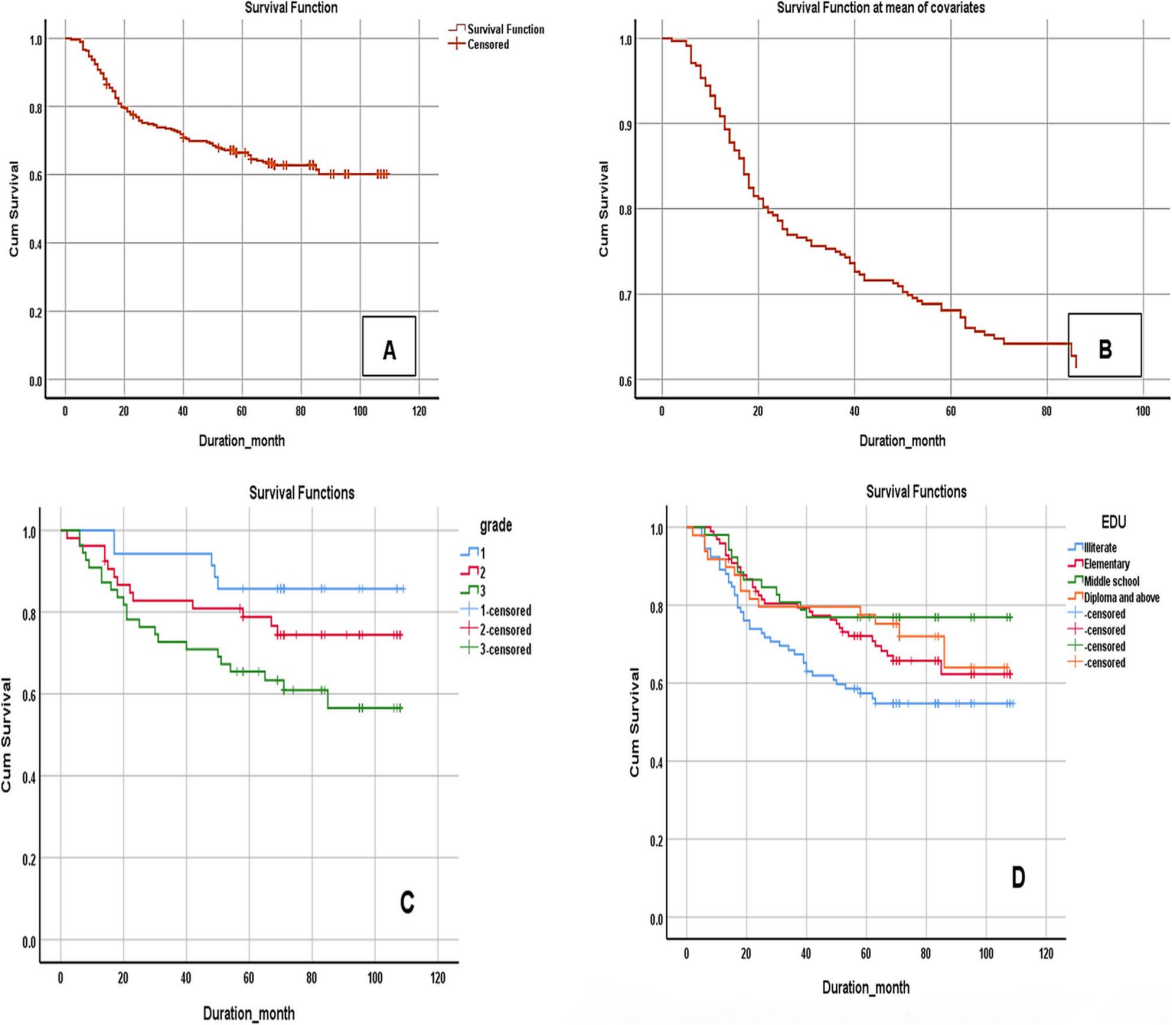
(**A**) Kaplan-Meier estimate of the cumulative survival of cervical cancer patients in Khuzestan, (2014 - 2019). (**B**) Cumulative survival of cervical cancer patients based on age at diagnosis. (**C**) Test of equality of survival distributions for the different levels of grade (Log rank:7.24, P = 0.027). (**D**)Test of equality of survival distributions for the different levels of education (Log rank,8.59 P = 0.provide caption)

The mean survival time is 77.47 (SD = 4.75 ; 95% CI:76.8–78.1) months during the years 2014 to 2019. The one-year, two-year, three-year, four-year and even five -year survival rates of cervical cancer cases are 89.8%, 76.9%, 73.2%, 69.5%, and 65.7%, respectively (Fig. 3A). A total of 34% of patients were lost to follow-up. Comparison of baseline characteristics between analyzed and censored patients showed no significant difference in mean age (*p*-value > 0.05). In the primary Kaplan–Meier analysis, the 5-year overall survival rate was 65.7%. To evaluate the potential influence of attrition bias, a sensitivity analysis was performed using mean imputed survival times of 26 months (average survival of deceased patients) and 70 months (average survival of living patients). Under these two extreme assumptions, the estimated 5-year survival rates were 51% (worst-case) and 70% (best-case), with the observed value (65.7%) lying between them, indicating reasonable robustness of the findings.

Among the variables examined in the univariate analysis, the age of patients at diagnosis (*p*-value = 0.009), the disease grade (*p*-value = 0.027), and the education level of patients have a significant effect on survival rate of cervical cancer patients (*p*-value = 0.035) (Fig. 3B, C, D and Table 2). Therefore, these variables were considered suitable candidates for inclusion in the multivariate Cox regression models. In current study, no significant relationship was found between employment status (*P*-value = 0.528) and ethnicity (*P*-value = 0.088) with survival rates, (Table 2).

According to the results of this analysis, only age and grade have a significant effect on survival rate, while education level did not show any significant relationship with survival in multivariate analysis, (Table 3 ). In this analysis, for each one-year increase in age, the risk of death increases by 4%. The risk of death in grades 3 and 2 is 3.5 and 2 times higher than grade 1, respectively (Table 3 ).

**Table 2:** The results of the univariate analysis test for comparing survival time in subgroups of independent variables in patients with cervical cancer in Khuzestan, Iran, (2014-2019)

	Variable		Mean of survival rate (month)	Std.Error	Log- Rank
Grade		Grade 1	98.6	4.42	7.24
		Grade2	87.89	5.02	
		Grade 3	75.86	5.52	
Education		Illiterate	71.27	4.51	8.59
		Elementary	81.57	3.85	
		Middle school	88.19	5.06	
		Diploma and above	82.97	5.57	
Employment status		housewife	80.02	2.46	0.39
		employed	8.92	9.77	
Ethnicity		Arab	77.04	3.92	6.54
		Bakhtiari	80.83	4.62	
		Lur	65.92	8.06	
		Fars	85.26	4.38	

**Table 3:** The results of the multivariate cox regression analysis aimed at examining the independent variables affecting the survival rate of patients with cervical cancer in Khuzestan (2014-2019)

Variable	Beta	Std.Error	*P* – Value	Hazard Ratio	95.0% CI for HR	
					Lower	Upper
Age	0.044	0.017	0.009	1.045	1.011	1.081
grade(1)	(reference)					
grade(2)	0.704	0.528	0.183	2.022	0.718	5.695
grade(3)	1.263	0.500	0.012	3.537	1.328	9.423
Diploma and above	(reference)					
Illiterate	− 0.555	0.544	0.307	0.574	0.198	1.667
Elementary	− 0.336	0.470	0.475	0.715	0.284	1.796
Middle school	− 0.621	0.575	0.280	0.537	0.174	1.659

## Discussion

Our findings indicate that, the trend of cervical cancer in Khuzestan province has been increasing (0.74 to 2.83 per 100.000) and it has decreased slightly from 2018 to 2019(2.83 to 2.81 per 100.000). Although the overall trend of the cervical cancer has been increasing over the years of study, Based on the results of the Chaichian study conducted from 2003 to 2009, the highest ASR (age standardized incidence rate) of Cervical cancer in Khuzestan Province was 4.48 per 100,000 people in 2008 [14]. Based on the results of our study, the highest ASR was 2.83 per 100,000 people in 2018. This indicates that over this ten-year period, the age-standardized incidence rate has significantly decreased in Khuzestan Province. Meanwhile, from 2003 to 2009, the national cancer registry was mainly pathologic based, the ASR of cervical was incremental which was higher than ASR in our study. In other words, ASR decreased from 2014 to 2019 as compared with 2003–2009 despite changing the registry system to a population-based cancer registry in the whole country [15, 16]. (ASR was reported to be 2.43, 4.48, and 3.69 per 100 000 in 2007, 2008, and 2009 and 2.65, 2.83 and 2.81 per 100000 in 2017, 2018 and 2019) [14]. In a study conducted by Anjam in 2016 across all provinces of Iran they also reached this conclusion and emphasized this point [16].It seems that there is a genuine decrease in the incidence of cervical cancer in Khuzestan as compared to the period from 2003 to 2009. This might be attributed to the increased public awareness and improved adherence to health practices. It maybe due to more referral of population for pap smear resulting in early detection of pre-cancerous lesions [17].

In the present study, the five-year survival rate of cervical cancer in the province during the years 2014 to 2019 was found to be 65.7%. According to the latest study conducted in Iran from 2008 to 2014, the five-year survival rate varied between 34% and 70% in different geographical areas, Khuzestan was among the provinces with the lowest five-year survival rates in the country (5-year SR 46%) [5]. This indicates that over these years, the survival rate in the province has increased significantly. The reported survival rate is similar to the rates of several European countries, South America, Southeast Asia, United States, and Russia, where the five-year survival rate ranges between 60% -70% [18]. Similarly, a meta-analysis conducted by Vali et al., in 2023, showed that the survival rate of patients with cervical cancer in Iran, Turkey, and Kuwait are higher compared to other countries in Asia [19]. Previous studies in Iran reported a five-year survival rate of 70.5% in Kerman Province (2012–2022) [20] and 70% in Hamadan Province (2008–2014) [5]. In our study, the five-year survival rate in Khuzestan was 65.7%. This lower rate may be related to more limited treatment facilities and a higher proportion of patients with grade 3 tumors in our study [21]. Despite the lack of organized screening in the province, the five - year survival rate has increased compared to the past, which may be linked to the sociocultural and sexual behavior and marriage rates [22]. In general, the survival rate of cervical cancer patients in this study is lower than other provinces in Iran [5]. The observed differences in survival may be attributed to factors such as the time of diagnosis, accuracy of staging, and appropriateness of treatment. In this context, prevention and early detection are critical. Iran currently lacks a national HPV vaccination program, and in Khuzestan, cost and access barriers further limit uptake, despite international evidence showing up to 90% reduction in cervical cancer incidence with vaccination [8, 23]. Similarly, national screening coverage remains below 45% [23], with cultural, geographic, financial, and literacy-related barriers (31.6% of our patients were illiterate). Implementing population-based HPV vaccination and organized, culturally adapted screening programs could improve early diagnosis and treatment, thereby reducing survival disparities [5, 20]. Although ethnicity was not statistically significant in the multivariable analysis, our results revealed a clinically important difference in survival, Lur patients had a mean survival of 66 months compared to 85 months for Fars patients (a20-month difference(. Potential explanations include differences in healthcare access, socioeconomic status, health literacy, and delays in diagnosis [19, 20]. These findings underscore the importance of targeted interventions for ethnic groups with limited access to care, despite the lack of statistical significance.

In the present study, Hazard ratio was found to be 3.5 in grade 3, whereas in a study conducted on patients with cervical cancer referred to cancer referral centers in Iran from 2014 to 2020, this rate was 3.1 in grade 3 and above, which is consistent with the current study [21]. A systematic review revealed that only around half of the Iranian women were aware of cervical cancer, and less than 45% of them had completed at least one lifetime Pap-smear test [24]. So, it is very important to note that early diagnosis of cervical cancer can significantly increase the chances of survival and recovery for patients. This requires the establishment of regular screening programs and raising awareness in various communities [25].

Based on the results of the Akbari study (2008–2014) the mean age of patients in Khuzestan province was 52.7 years. Whereas in the current study, the mean age of patients was found to be 54 years. This indicates that over the years, the mean age of patients in the province has remained relatively stable. Additionally, the mean age of patients in Iran is slightly higher than the global mean age (52.2 years) [5], and higher than several European countries (44.7 years) [7]. It may be due to differences in cultural and religious beliefs, as most Muslim women living in Asia and Iran restrain from having sexual intercourse before marriage and limit their sexual activity to only one partner after marriage [26]. Additionally, cultural differences may be more pronounced in Khuzestan [12]. These differences may explain why these countries have lower cervical cancer incidence and higher mean age of patients than Western countries [6, 22, 26]. The highest age-specific incidence rate of cervical cancer in Khuzestan province has been observed in the age group of 80 years and above. The causes for this increase can be due to lack of patient referrals and limited access to diagnostic facilities for the elderly women [14]. Another reason could be the smaller population of women in older age groups; even one or a few cases of cancer can significantly impact the age-specific incidence rate [27]. although Numerous studies have documented that the risk of cervical cancer increases with advancing age, particularly beyond 65 years. For instance, more than 20 ٪ of cervical cancer cases occur in women aged 65 or older, and in several populations the incidence in this age group remains substantial findings that reinforce the need to interpret our observed high incidence in the ≥ 80 age group in light of age-related vulnerability [28, 29].

In the present study, most of the cervical cancer patients were housewives (93.5%) and illiterate or had only primary education (64%). A similar study was conducted in India revealed that more than half of the cervical cancer patients (53.6%) were illiterate, and only 3.66% had a university education. Most patients were housewives (86.14%), and 3.92% of women were employed [30]. This finding aligns with many studies that consider illiteracy can also be a risk factor for cervical cancer [31]. So, it is important to note that raising awareness and public information about the risk factors for cervical cancer, as well as educational and preventive programs, regular screenings, and vaccinations (such as the HPV vaccine), is very crucial in communities with high illiteracy rates and any approach taken needs to take this into account .

## Limitations

This study has several important limitations. The most critical is attrition bias, as 34% of patients were lost to follow-up, which may have biased survival estimates upward or downward depending on the outcomes of those lost. Although sensitivity analyses indicated robustness, this bias cannot be fully excluded.

Other limitations include limited follow-up time for patients diagnosed in 2018–2019, possibly introducing lead-time bias, as some of these patients had not yet reached the five-year observation point; the absence of TNM staging, treatment, and screening data, which restricted identification of prognostic factors; and a six-year study period insufficient for reliable trend analysis during registry transitions. Additionally, generalizability is limited due to Khuzestan’s distinct demographic and healthcare characteristics.

Minor limitations involve potential underreporting or misclassification of cases, as well as incomplete contact information and possible recall bias in mortality data. Future studies should integrate multiple data sources and link registry data with national vital statistics to improve accuracy and completeness.

## Conclusion

According to the current study, the incidence of cervical cancer in Khuzestan province has been increasing during this six-year period (2014–2019), although this rate has decreased compared to previous years. Additionally, while the survival rate in the province has improved, it is still lower than in other provinces in Iran. Furthermore, the level of illiteracy among patients is high, and illiteracy is considered a risk factor for cervical cancer. Cultural issues and behavioral factors are very important in Khuzestan province, and social factors primarily affect the incidence and survival rates of cervical cancer. Implementation of effective screening and preventive programs that take into account cultural and socio-economic issues, along with continuous monitoring of survival outcomes and identification of high-risk groups for resource allocation, is recommended.

## Data Availability

The datasets used and/or analyzed during the study will be made available by the corresponding author following a reasonable request.

## Abbreviations

ASR: Age-standardized incidence rate
ASIR: Age-specific incidence rate
CR: Crude rates
SR: Survival rate
5-year SR: Five-year survival rate
ICD-O-3: International classification of diseases for oncology, third edition
HPV: Human papilloma virus
MAPE: Mean absolute percentage error
